# An Exploratory Analysis of Differential Prescribing of High-Risk Opioids by Insurance Type Among Patients Seen by the Same Clinician

**DOI:** 10.1007/s11606-023-08025-6

**Published:** 2023-02-06

**Authors:** Lucy B. Schulson, Andrew Dick, Flora Sheng, Bradley D. Stein

**Affiliations:** 1 RAND Corporation, Boston, MA USA; 2 Boston University Aram V. Chobanian & Edward Avedisian School of Medicine, Boston, MA USA; 3 Columbia University School of Nursing, New York, NY USA; 4 RAND Corporation, Arlington, VA USA; 5 RAND Corporation, Pittsburgh, PA USA; 6 Medicine, University of Pittsburgh, Pittburgh, PA USA

**Keywords:** opioids, insurance, disparities

## Abstract

**Background:**

Insurance status may influence quality of opioid analgesic (OA) prescribing among patients seen by the same clinician.

**Objective:**

To explore how high-risk OA prescribing varies by payer type among patients seeing the same prescriber and identify clinician characteristics associated with variable prescribing

**Design:**

Retrospective cohort study using the 2016–2018 IQVIA Real World Data – Longitudinal Prescription

**Participants:**

New OA treatment episodes for individuals ≥ 12 years, categorized by payer and prescriber. We created three dyads: prescribers with ≥ 10 commercial insurance episodes and ≥ 10 Medicaid episodes; ≥ 10 commercial insurance episodes and ≥ 10 self-pay episodes; and ≥ 10 Medicaid episodes and ≥ 10 self-pay episodes.

**Main Outcome(s) and Measure(s):**

Rates of high-risk episodes (initial opioid episodes with > 7-days’ supply or prescriptions with a morphine milliequivalent daily dose >90) and odds of being an unbalanced prescriber (prescribers with significantly higher percentage of high-risk episodes paid by one payer vs. the other payer)

**Key Results:**

There were 88,352 prescribers in the Medicaid/self-pay dyad, 172,392 in the Medicaid/commercial dyad, and 122,748 in the self-pay/commercial dyad. In the Medicaid/self-pay and the commercial-self-pay dyads, self-pay episodes had higher high-risk episode rates than Medicaid (16.1% and 18.4%) or commercial (22.7% vs. 22.4%). In the Medicaid/commercial dyad, Medicaid had higher high-risk episode rates (21.1% vs. 20.4%). The proportion of unbalanced prescribers was 11–12% across dyads. In adjusted analyses, surgeons and pain specialists were more likely to be unbalanced prescribers than adult primary care physicians (PCPs) in the Medicaid/self-paydyad (aOR 1.2, 95% CI 1.16–1.34 and aOR 1.2, 95% CI 1.03–1.34). For Medicaid/commercial and self-pay/commercial dyads, surgeons had lower odds of being unbalanced compared to PCPs (aOR 0.6, 95% CI 0.57–0.66 and aOR 0.6, 95% CI 0.61–0.68).

**Conclusions:**

Clinicians prescribe high-risk OAs differently based on insurance type. The relationship between insurance and opioid prescribing quality goes beyond where patients receive care.

**Supplementary Information:**

The online version contains supplementary material available at 10.1007/s11606-023-08025-6.

## INTRODUCTION

Patients with different insurance types often receive care of different quality.^1–9^ Variation in quality often stems from differences in where patients receive care.^10–12^ For example, hospitals with a high proportion of Medicaid patients have lower adherence to quality measures than hospitals with fewer Medicaid patients.^13^ However, variability in care quality does not stem solely from differences between providers. Indeed, it varies among patients treated in the same hospital system^2^ or by the same clinician.^14^

Recent studies suggest that quality of opioid analgesic (OA) prescribing may also vary by insurance type. Patterns of OA prescribing such as high-risk or potentially inappropriate prescribing and rapid discontinuation are considered potential indicators of poor quality, given their association with harms including an increased risk of overdose, development of opioid use disorder, poorer health outcomes, and a greater likelihood of remaining out of the workforce.^15–21^ High-risk OA prescribing, such as initial prescriptions >7 days or >90 morphine milligram equivalents (MME), varies by insurance status: compared to the commercially insured, Medicaid and self-pay patients experience high-risk prescribing at higher rates.^22^ Compared to commercially insured patients, Medicaid enrollees and self-pay patients receiving long-term opioids are more likely to have episodes rapidly discontinued.^23^ Self-pay patients on long-term opioids are also less likely to receive naltrexone co-prescriptions compared to those with insurance.^24^

Some variability in opioid prescribing patterns likely reflects differences in *where* patients receive care—e.g., in health systems or from prescribers more likely to provide lower quality opioid care.^25^ For example, approximately 1% of prescribers account for 49% of all opioid doses prescribed in the US, and they commonly prescribe higher opioid doses than recommended by the Centers for Disease Control and Prevention (CDC).^26^ Some clinicians are also more likely to prescribe high-risk opioids than others,^27^ even when working in the same health system.^28^

However, OA prescribing quality may vary because prescribers treat patients with different insurance statuses differently. A study of access to medication treatment for opioid use disorder for pregnant individuals found significant variation within the same practice based on insurance status.^29^ However, we are unaware of studies examining to what extent insurance status may influence the quality of OA prescribing by the same clinician or what the characteristics are of clinicians more likely to prescribe differently based on insurance status.

To address this gap in the literature, we used national pharmacy claims from approximately 90% of US retail pharmacies to conduct an exploratory analysis identifying prescribers treating at least twenty individuals with OA prescriptions during the period January 2017 through December 2018. We examined to what extent prescribers may have different rates of high-risk OA prescribing to patient populations with different insurance statuses. We also examined provider specialty and its association with high-risk prescribing to patients with different insurance statuses. State, federal, and local stakeholders seeking to decrease high-risk OA prescribing^30,31^ need to know if prescribers prescribe differently based on a patient’s insurance status. Policy interventions beyond those commonly implemented would be essential if certain patient populations are more likely to receive high-risk opioid prescriptions, even when treated by the same clinician.

## METHODS

### Analytic Dataset

We used de-identified pharmacy claims from October 1, 2016, through December 31, 2018, from the IQVIA Real World Data – Longitudinal Prescription^32^ to identify new opioid treatment episodes initiated between January 1, 2017, and October 1, 2018, for individuals aged 12 years and older. IQVIA data have been used in multiple studies of opioid analgesic prescribing,^22–24,33–35^ and capture an estimated 90% of all prescriptions dispensed at retail pharmacies in all 50 states and the District of Columbia. The data include information on the prescription, payer, some patient demographics, prescriber specialty and location, each dispensed prescription’s natural drug code, dosage, quantity, and days’ supply. Patients have unique identifiers enabling researchers to identify prescriptions dispensed to that individual across pharmacies. We excluded buprenorphine formulations (e.g., suboxone) primarily used to treat opioid use disorder.

**Figure 1:**
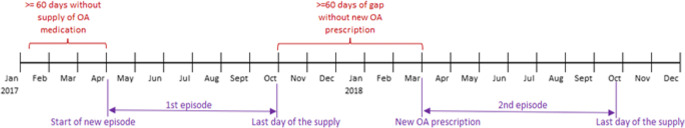
Illustration of creation of opioid episodes.

We defined new treatment episodes as the first observed date of a dispensed opioid prescription following a period of at least 60 days after the days’ supply of any prior dispensed opioid prescription had run out. An episode lasted through the last day of the supply for the last dispensed opioid prescription, with no more than a 60-day gap between the last day supply of one prescription and the subsequent prescription being dispensed (Fig. 1). We limited our sample to episodes from prescribers who wrote OA prescriptions for at least twenty individuals during the 2 years. We categorized each episode by payer and prescriber of the first prescription and identified three groups of prescribers: prescribers with (1) ≥ 10 commercial insurance episodes and ≥ 10 Medicaid episodes; (2) ≥ 10 commercial insurance episodes and ≥ 10 self-pay episodes; and (3) ≥ 10 Medicaid episodes and ≥ 10 self-pay episodes. We label these provider groups respectively as (1) Medicaid/commercial, (2) commercial/self-pay, and (3) Medicaid/self-pay. A prescriber could be in more than one payer dyad.

### Measures

#### High-Risk Prescribing

Focusing on an episode’s first prescription, we identified high-risk prescribing practices associated with poor clinical outcomes such as increased risk of chronic opioid use, overdose, and opioid-related mortality.^36–39^ Specifically, we identified initial opioid prescriptions with (1) greater than a 7-day supply, or (2) high-dose prescriptions with a MME daily dose >90, using the CDC MME conversion factor.^40^

#### Prescriber Specialty and Provider Types

We categorized prescribers into six specialty/provider type groups: adult primary care physicians (including general internists and family practice physicians); advance practice prescribers (nurse practitioners and physician assistants, APP); surgeons; emergency physicians; anesthesiologists, pain specialists, and neurologists (hereafter pain specialists); and other specialties (including pediatricians, internal medicine subspecialists).

#### County Characteristics

We controlled for county characteristics suggested in prior research as potentially influencing opioid prescribing^22,23,41^ as well as for the total amount of opioid per capita dispensed in the county. We used a 5-digit FIPS code to determine the county in which a prescriber practiced. We included community characteristics including the non-White percent of population from the US Census Bureau;^42^ “urban” (RUCC 1, 2, or 3) or “rural” (RUCC 4 and greater) urbanicity based on Rural-Urban Continuum Codes (RUCC);^43^ county overdose rates per capita using CDC data;^44^ and total opioid per capita as a population-weighted measure, calculated using days’ supply and total daily opioid dose from all opioid prescriptions filled in a county, and categorized into terciles based on annual distribution.

### Analytic Approach

We developed four mutually exclusive and exhaustive prescriber groups of prescribers based on the overall rate of high-risk prescribing (pooled across arms of the dyad) for each prescriber and a test statistic of the null hypothesis that the difference between rates of high-risk prescribing across arms was 0. We defined the provider groups as (1) *balanced prescribers*—those with low overall rates of high-risk prescribing (lower than the overall mean of high-risk prescribing across all prescribers in the dyad and small differences in the rates of high-risk prescribing across arms of the dyad, difference statistic within the 20% confidence interval around 0 [*p*-value >0.80]); (2) *moderate prescribers*—those with low overall rates of high-risk prescribing and moderate differences in rates of high-risk prescribing across arms of the dyad (difference statistic outside of the 20% confidence interval around 0 but also not within a rejection region defined by alpha=0.10 [*p*-value <0.80 and *p*-value >0.10]); (3) *unbalanced prescribers*—those who had significant differences in rates of prescribing across dyad arms (difference statistic fell within the 10% rejection region [*p*-value <0.10]); and (4) *consistent high-risk prescribers*—those who had high overall rates of high-risk prescribing and no more than moderate differences between rates of high-risk prescribing between dyad arms (difference statistic outside of the rejection region [*p*-value >0.10]).

For each payer group, we performed bivariate analyses to identify associations between prescriber status (balanced, moderate, unbalanced, high-risk) and prescriber and county characteristics. To identify factors associated with being an unbalanced prescriber, we used multivariable regression to compare unbalanced prescribers to all other prescribers, by specialty and controlling for county urbanicity, opioid per capita dispensed, fatal overdose rate, and percent of non-White residents. We controlled for minimum log-transformed volume of patients of each prescriber receiving opioids to correct uncertainty in the unbalanced group that could be introduced by disproportionately high-volume prescribers. We included state fixed effects and robust standard errors clustered at the county level to account for unobserved correlation structures across observations within a county.^45,46^ We performed bivariate analyses of patient characteristics by episode overall and by prescriber status for each dyad, and conducted sensitivity analyses where high-dose prescriptions were defined as MME daily dose >90. The study was approved with a waiver of consent by the corresponding author’s IRB.

## RESULTS

Our analyses included 157,548,144 prescriptions accounting for 80.1% OA prescriptions dispensed during the study period. The 88,352 clinicians in the Medicaid/self-pay dyad were responsible for 8,078,116 episodes; the 172,392 clinicians in the Medicaid/commercial dyad were responsible for 20,148,616 episodes; the 122,748 clinicians in the self-pay/commercial dyad were responsible for 16,676,807 episodes (Table 1).

**Table 1:** High-Risk^*^ Prescribing Episode Rates, by Dyad

Prescriber dyad: Medicaid and self-pay (*n*=73,283)^†^	
High-risk prescribing rates in episodes treated^*^	%
Medicaid (*n*=5,028,557 episodes)	16.1
Self-pay (*n*=3,049,559 episodes)	18.4
Fraction of prescribers in prescriber dyad	%
Self-pay rate > Medicaid rate	49.8
Self-pay rate = Medicaid rate	20.8
Self-pay rate < Medicaid rate	29.4
Distribution of prescribers in each prescribing category	
Balanced^‡^	33.6
Moderate^§^	54.5
Unbalanced^‖^	11.8
High-risk ^¶^	0.04
Prescriber dyad: Medicaid and commercial (*n*=138,783) ^†^	
High-risk prescribing rates in episodes treated^*^	%
Medicaid (*n*=13,021,015 episodes)	21.1
Commercial (*n*= 7,127,601 episodes)	20.4
Fraction of prescribers in prescriber dyad	%
Medicaid rate > commercial rate	42.0
Medicaid rate = commercial rate	14.6
Medicaid rate < commercial rate	43.4
Distribution of prescribers in each prescribing category	%
Balanced^‡^	28.7
Moderate^§^	60.1
Unbalanced^‖^	11.1
High-risk^¶^	0.1
Prescriber dyad: commercial and self-pay (*n*=105,192)^†^	
High-risk prescribing rates in episodes treated^*^	%
Self-pay (*n*=3,741,807 episodes)	22.7
Commercial (*n*=12,935,000 episodes)	22.4
Fraction of prescribers in prescriber dyad	%
Self-pay rate > commercial rate	55.3
Self-pay rate = commercial rate	14.2
Self-pay rate < commercial rate	30.5
Distribution of prescribers in each prescribing category	%
Balanced^‡^	29.0
Moderate^§^	59.2
Unbalanced^‖^	11.8
High-risk^¶ ¶^	0.1

^*^High-risk prescribing is either initial prescription > 7 days or prescription for greater than 90 milliequivalents of morphine

^†^*n* is number of providers in dyad

^‡^Balanced prescribers: clinicians whose overall high-risk prescribing was less than the mean pooled rate among all in the dyad and for whom the probability that the rates of high risk and difference between the rates of high-risk prescribing across payers are within a 20% confidence interval around zero

^§^Moderate prescribers: clinicians for whom the rate difference in high-risk prescribing between payers fell between 0 and the top 5th or 0 and the bottom 5th percentile of the distribution

^‖^Unbalanced: clinicians whose prescribing rate difference in prescribing between insurances was either in the top or bottom 5th percentile

^¶^High-risk prescribers: clinicians whose overall high-risk prescribing was higher than mean pooled rate among all in the dyad

Among the Medicaid/self-pay dyad, 18.4% of self-pay episodes involved high-risk prescriptions compared to 16.1% of Medicaid episodes. Almost half (49.8%) prescribers wrote high-risk prescriptions at higher rates to self-pay patients than Medicaid patients, 29.4% wrote at higher rates to Medicaid patients than self-pay patients, and 20.8% wrote at comparable rates between the two groups. In the self-pay/commercial dyad, 22.7% of self-pay episodes and 22.4% of commercial episodes involved high-risk prescriptions. Of prescribers in this dyad, 55.3% wrote at higher rates of high-risk prescriptions to self-pay patients than commercial patients, 30.5% wrote at higher rates to commercial patients than self-pay patients, and 14.2% wrote at comparable rates between the two groups. In the Medicaid/commercial dyad, 21.1% of Medicaid episodes and 20.4% of commercial episodes involved high-risk prescriptions. Of prescribers in the dyad, 42.0% wrote at higher rates of high-risk prescriptions to Medicaid patients than commercial patients, 43.4% wrote at higher rates to commercial patients than Medicaid patients, and 14.6% had comparable rates between the two groups (Table 1).

Of clinicians in the Medicaid/self-pay dyad, 33.6% were balanced prescribers, 54.5% were moderate prescribers, 11.8% were unbalanced prescribers, and 0.04% were consistently high-risk prescribers. Distributions in the Medicaid/commercial and self-pay/commercial dyads were similar: balanced (28.7% and 29.0%); moderate (60.1% and 59.2%); unbalanced (11.1% and 11.8%); and high-risk (0.1% and 0.1%, respectively) (Table 1).

PCPs (25.5%), APPs (24.7%), and emergency physicians (24.8%) comprised the majority of clinicians in the Medicaid/self-pay dyad; only 2.4% were pain physicians. The majority of unbalanced prescribers in this dyad were PCPs and surgeons (34.7% and 32.2%, respectively). A large proportion of unbalanced prescribers were located in counties with a high proportion of minority populations (49.2%); few were in counties with the lowest overdose rates (6.6% in first quartile overdose rate per capita). Unbalanced prescribers were predominantly in urban settings (82.5%) and in regions with high opioids per capita (58.0% in third tercile of opioids per capita) (Table 2).

**Table 2:** Characteristics of Prescribers, Overall and Unbalanced, by Dyad

	Prescriber dyad: Medicaid and self-pay^*^		Prescriber dyad: Medicaid and commercial^*^		Prescriber dyad: self-pay and commercial^*^	
Prescriber characteristics	Overall dyad^**†**^ *n*=88,352	Unbalanced *n*=10,447	Overall dyad^**†**^ *n*=172,391	Unbalanced *n*=19207	Overall dyad^**†**^ *n*=122,748	Unbalanced *n*=14,521
	%	%	%	%	%	%
Specialty/Provider type						
Primary Care Physician	25.5	34.7	32.5	39.8	27.6	38.4
Emergency Physician	24.8	5.1	15.8	3.4	19.6	8.2
Other specialty	3.7	4.0	4.4	7.1	4.2	6.1
Physician Assistant/Nurse Practitioner	24.7	20.3	24.8	26.8	22.7	22.1
Pain Physician	2.4	3.8	2.6	4.0	3.1	4.0
Surgeon	19.0	32.2	19.9	18.9	22.9	21.2
Quartile non-White or Hispanic						
First (most White)	5.1	5.5	6.0	7.0	4.4	5.3
Second	12.7	13.3	14.0	13.2	11.6	11.2
Third	30.7	32.1	32.2	31.0	30.3	28.9
Fourth (most minority)	51.5	49.2	47.8	48.8	53.7	54.6
Overdose rate (quartile)						
First (lowest rate)	7.4	6.6	6.1	5.4	7.9	8.6
Second	29.9	26.5	25.1	23.1	32.5	32.2
Third	33.1	37.6	31.3	32.8	32.2	31.9
Fourth (highest rate)	29.6	29.2	37.5	38.6	27.4	27.3
Urbanicity						
Urban	82.9	82.5	84.6	84.2	84.0	80.9
Rural	17.1	17.5	15.4	15.8	16.0	19.1
Total Opioid per capita (tercile)						
First (lowest)	9.0	7.7	9.4	9.8	8.8	10.2
Second	37.8	34.3	39.5	38.3	38.5	37.0
Third	53.3	58.0	51.1	51.9	52.7	52.8

^*^We excluded a few providers whose rurality status cannot be determined due to lack of data for analytic purposes

^†^Chi-square tests comparing characteristics of unbalanced, balanced, moderate, and high-risk prescribers were performed for each dyad. All *p*-values were <.0001. *p*-value < .05 is statistically significant

Results were similar for the Medicaid/commercial dyad, except the most common unbalanced prescribers in this dyad were PCPs and APPs (39.8% and 26.8%, respectively). In the self-pay/commercial dyad, the most common prescribers were PCPs and surgeons (27.6% and 22.9%), and the most common unbalanced prescribers in this dyad were PCPs and APPs (38.4% and 22.1%) (Table 2). Characteristics of balanced, moderate, and high-risk prescribers were similar across dyads (Appendix 1).

In the multivariable analyses, we examined factors associated with being an unbalanced prescriber. In the Medicaid/self-pay dyad, we found that compared to adult PCPs, pain physicians and surgeons were significantly more likely to be unbalanced prescribers (aOR 1.2, 95% CI 1.03–1.34 and aOR 1.2, 95% CI 1.16–1.34). Emergency physicians, other specialties, and APPs had lower odds of being unbalanced prescribers (aOR 0.2, 95%CI 0.18–0.23; aOR 0.9, 95% CI 0.77–0.98; aOR 0.6, 95%CI 0.59–0.68). Results were similar for the Medicaid/commercial and self-pay/commercial dyads, except that surgeons had a lower odds of being unbalanced compared to PCPs (aOR 0.6, 95%CI 0.57–0.66; aOR 0.6, 95%CI 0.61–0.68) and other specialties had higher odds (aOR 1.2, 95%CI 1.08–1.28; aOR 1.2, 95% CI 1.10, 1.29) (Table 3). None of the dyads showed clear patterns between county characteristics and unbalanced prescribing. Patient characteristics within dyads were similar across prescriber groups (Appendix 2). Sensitivity analyses with high-risk OA prescriptions limited to those with >90 MME yielded similar results (Appendix 3).

**Table 3:** Adjusted Odds Ratio^*^ of Characteristics of Unbalanced Prescribers vs. Other Prescribers^†^

	Medicaid-self-pay dyad aOR^‡^ (95% CI)	Medicaid-commercial dyad aOR^‡^ (95% CI)	Commercial-self-pay dyad aOR^‡^ (95% CI)
Specialty/Provider type			
Primary Care Physician	Ref	Ref	Ref
Emergency Physician	**0.2 (0.18–0.23)**	**0.2 (0.19–0.24)**	**0.5 (0.44–0.52)**
Other specialty	**0.9 (0.77–0.98)**	**1.2 (1.08–1.28)**	**1.2 (1.1–1.29)**
Physician Assistant/Nurse Practitioner	**0.6 (0.59–0.68)**	**0.8 (0.8–0.89)**	**0.8 (0.74–0.83)**
Pain Physician	**1.2 (1.03–1.34)**	**1.3 (1.21–1.49)**	1 (0.87–1.07)
Surgeon	**1.2 (1.16–1.34)**	**0.6 (0.57–0.66)**	**0.6 (0.61–0.68)**
Quartile non-White or Hispanic			
First	Ref	Ref	Ref
Second	0.9 (0.82–1.08)	0.9 (0.8–0.95)	**0.9 (0.77–0.97)**
Third	1 (0.86–1.12)	0.9 (0.86–1.04)	0.9 (0.81–1.02)
Fourth	1 (0.87–1.17)	**1.2 (1.05–1.33)**	1.1 (0.94–1.23)
Overdose rate (quartile)			
First	Ref	Ref	Ref
Second	1 (0.85–1.1)	1.1 (0.94–1.19)	1 (0.92–1.11)
Third	1.1 (0.93–1.23)	1.1 (0.93–1.19)	1 (0.88–1.1)
Fourth	1 (0.87–1.18)	**1.2 (1.04–1.38)**	1 (0.91–1.18)
Urbanicity			
Urban	Ref	Ref	Ref
Rural	1 (0.91–1.07)	**1.2 (1.11–1.27)**	**1.4 (1.26–1.45)**
Total opioid per capita (tercile)			
First	Ref	Ref	Ref
Second	1 (0.88–1.21)	1 (0.88–1.12)	**0.9 (0.8–0.95)**
Third	1.1 (0.91–1.26)	1.1 (0.95–1.2)	0.9 (0.86–1.02)
Minimum patient volume per prescriber^‡^	**0.1 (0.06–0.08)**	**0.2 (0.17–0.2)**	**0.1 (0.11–0.14)**

*Logistic regression models also included state fixed effects. Standard errors were clustered at the county level

†Comparison group includes balanced, moderate, and high-risk prescribers

‡Log-transformed minimum number of patients across 2 payers of each provider

Odds ratios that are statistically signification at *p* <.05 are bolded

## DISCUSSION

More than 10% of clinicians in our sample were significantly more likely to write high-risk prescriptions (more than 7-day supply or more than 90 MME) to patients of one insurance type compared to their patients of another insurance type. Such high-risk prescribing was more common for self-pay patients than for commercially insured and Medicaid-enrolled patients among clinicians treating both patient populations, suggesting that the relationship between insurance status and quality of prescribing OAs goes beyond where patients receive care. This differential prescribing may stem from factors such as patient education or patient demographics not captured in the data. Nonetheless, our study suggests that insurance type itself is a marker for risk of lower quality opioid care. Prior studies have shown that access to health insurance improves opioid-related health outcomes, probably reflecting access to opioid use disorder treatment.^47^ We found that access to insurance—either Medicaid or commercial—is associated with safer initial prescribing of OAs.

We found that self-pay patients had more high-risk opioid episodes than Medicaid or commercial patients but can only hypothesize about the reason. Clinicians may alter their practices due to clinical differences among populations, implicit or explicit bias, or misaligned financial incentives.^48^ For example, commercially insured patients may have fewer high-risk opioid episodes because they are receiving other treatments, such as surgery or physical therapy. These patients may be more likely than Medicaid patients to receive such interventions because of higher reimbursement^48^ or because such procedures may be prohibitively expensive for self-pay patients. Patients who cannot afford alternative treatments may be more reliant on opioids, and it may be more cost-effective for such patients to receive a shorter course of higher dose opioids than a longer course of lower dose opioids. Clinicians may be responding to these realities.

Unbalanced prescribing may reflect prescribing restrictions for patients with commercial and Medicaid insurance but not for self-pay. Alternatively, because many commercial insurers and Medicaid programs monitor and track opioid prescriptions using pharmacy claims,^49^ some individuals who self-pay for opioids may be seeking to evade or circumvent such efforts.^50,51^ It is also possible that self-pay patients who received an initial episode >7 days were in fact on long-term opioids but had gaps in prescriptions because they were unable to pay for medications. Finally, prescribers themselves may also suggest self-payment to evade the need for prior authorization or prescription monitoring.

Studies show that high-risk prescribing varies by clinician type and characteristics.^52^ However, results are mixed regarding which prescriber specialties are more likely to write high-risk prescriptions. Fink and colleagues found patients of nurse practitioners (NP) or naturopathic medicine clinicians received more high risk opioids prescriptions than patients seen by other clinician types. However, these high-risk prescriptions were actually written by prescribers in different disciplines (i.e., not their NP or naturopathic medicine clinician). These patients were just more likely to have multiple prescribers, and the authors concluded that variability was due to patient rather than prescriber characteristics.^53^ A study of post-surgical opioid prescribing found APPs were more likely to prescribe opioids >30 days than physicians across all surgical specialties.^54^ By contrast, studies in the emergency department found that APPs were less likely to prescribe high-risk opioids.^55^ Another study found PCPs were more likely to prescribe opioids than specialists, but surgeons and hospital-based specialists prescribed higher doses than PCPs.^52^ However, no prior work has examined the extent to which individual clinicians prescribe differently to different patients.

We found that pain physicians had greater odds of being unbalanced prescribers than PCPs. Patients seeking care with pain specialists may have chronic, more complex pain than patients seeing other prescribers. However, it is possible that self-pay patients who obtain care from pain specialists differ clinically from commercially insured or Medicaid-enrolled individuals receiving such care. For example, self-pay patients may present to pain care clinicians later in the course of their illness, potentially contributing to higher rates of high-dose opioids or longer prescriptions observed in self-pay individuals.

### Limitations

Our study has several limitations. We have no clinical information about individuals filling opioid prescriptions. Without clinical information, we do not know to what extent the high-risk prescribing is appropriate (e.g., palliative treatment), or whether clinical differences are driving variation between populations. We have no information about opioids dispensed from pharmacies not included in the IQVIA data, or information about other services received by individuals filling opioid prescriptions, including non-pharmacologic interventions for pain management. We have no information about the clinical settings in which prescribers practice. Many clinicians work in multiple settings, potentially treating individuals with different insurance in different settings, thereby influencing opioid prescribing. Finally, although we have developed a concrete, robust, and reproducible method for classifying providers as unbalanced, the approach is novel, and we therefore cannot compare our characterizations with those used elsewhere. Future research examining such characterizations is needed.

## CONCLUSION

Our study enhances understanding of potentially high-risk prescribing of OAs. The same clinicians prescribed OAs differently to different populations of patients. Over 1 in 10 prescribers in each of the dyads exhibited unbalanced prescribing patterns based on insurance type, and self-pay patients were particularly at risk for receiving high-risk opioid episodes. Our findings may reflect barriers to access to care for patients without insurance, clinician efforts to limit out-of-pocket costs to uninsured patients, or limitations of current opioid prescribing monitoring systems. Our findings warrant further examination of how the structure of the US health system may contribute to disparate high-risk prescribing. Specifically, we need a better understanding of the extent to which our findings reflect barriers to access to care for patients without insurance and clinician efforts to limit out-of-pocket costs to uninsured patients. These results may also reflect the limitations of current opioid prescribing monitoring systems and the need for them to better capture patients who pay out-of-pocket for their medications. Our findings may further support the need for health insurance expansion and additional policy interventions to address the disparities observed in potentially high-risk opioid prescribing by the same clinician to different populations of patients.

## Supplementary Information

ESM 1 (DOCX 37 kb)
