# Supplementary material for: An Exploratory Analysis of Differential Prescribing of High-Risk Opioids by Insurance Type Among Patients Seen by the Same Clinician
Source: J Gen Intern Med. 2023 Feb 6;38(7):1681–8. doi: 10.1007/s11606-023-08025-6 (PMC10212884; doi:10.1007/s11606-023-08025-6)
Supplement: Supplementary file 1 — (DOCX 37 kb) [file 11606_2023_8025_MOESM1_ESM.docx]

**Appendix Table 1: Prescriber Characteristics in each dyad and within prescribing groups: balanced, moderate, and high-risk**

| **Prescriber Dyad: Medicaid and Self-pay** | | | |
| --- | --- | --- | --- |
| **Prescriber characteristics** | **Balanced group* n=29,727** | **Moderate^†^ n=48,141** | **High-risk prescribers^‡^ n=37** |
| **Specialty/Provider type** | **%** | **%** | **%** |
| Primary Care Physician |  |  |  |
| Emergency Physician | 14.5 | 30.2 | 18.9 |
| Other specialty | 43.3 | 17.7 | 0.0 |
| Physician Assistant/ Nurse Practitioner | 3.5 | 3.7 | 0.0 |
| Pain Physician | 31.9 | 21.1 | 21.6 |
| Surgeon | 0.2 | 3.5 | 56.8 |
| **Quartile Non-white or Hispanic** | 6.5 | 23.8 | 2.7 |
| First (most white) |  |  |  |
| Second | 4.7 | 5.3 | 8.1 |
| Third | 12.8 | 12.5 | 16.2 |
| Fourth (most minority) | 31.4 | 30.0 | 16.2 |
| **Overdose rate (quartile)** | 51.1 | 52.3 | 59.5 |
| First (lowest rate) |  |  |  |
| Second | 6.1 | 8.4 | 5.4 |
| Third | 27.1 | 32.4 | 35.1 |
| Fourth (highest rate) | 33.6 | 31.8 | 27.0 |
| **Urbanicity** | 33.2 | 27.4 | 32.4 |
| Urban |  |  |  |
| Rural | 86.5 | 80.9 | 86.5 |
| **Total Opioid per capita (tercile)** | 13.5 | 19.1 | 13.5 |
| First (lowest) |  |  |  |
| Second | 8.6 | 9.5 | 10.8 |
| Third | 39.5 | 37.5 | 18.9 |
| **Prescriber Dyad: Medicaid and Commercial** | | | |
| **Prescriber characteristics** | **Balanced group* n=49,535** | **Moderate^†^ n=103,525** | **High-risk prescribers^‡^ n=124** |
| **Specialty/ Provider type** | **%** | **%** | **%** |
| Primary Care Physician |  |  |  |
| Emergency Physician | 19.0 | 37.6 | 15.3 |
| Other specialty | 36.0 | 8.5 | 0.0 |
| Physician Assistant/ Nurse Practitioner | 3.9 | 4.1 | 4.8 |
| Pain Physician | 28.6 | 22.6 | 30.6 |
| Surgeon | 0.2 | 3.5 | 48.4 |
| **Quartile Non-white or Hispanic** | 12.3 | 23.8 | 0.8 |
| First (most white) |  |  |  |
| Second | 5.4 | 6.2 | 2.4 |
| Third | 13.9 | 14.2 | 5.6 |
| Fourth (most minority) | 32.7 | 32.1 | 35.5 |
| **Overdose rate (quartile)** | 48.0 | 47.5 | 56.5 |
| First (lowest rate) |  |  |  |
| Second | 5.2 | 6.6 | 2.4 |
| Third | 23.2 | 26.4 | 28.2 |
| Fourth (highest rate) | 31.9 | 30.7 | 37.9 |
| **Urbanicity** | 39.7 | 36.3 | 31.5 |
| Urban |  |  |  |
| Rural | 87.1 | 83.5 | 87.1 |
| **Total Opioid per capita (tercile)** | 12.9 | 16.5 | 12.9 |
| First (lowest) |  |  |  |
| Second | 8.8 | 9.6 | 3.2 |
| Third | 40.7 | 39.1 | 28.2 |
| **Prescriber dyad: Commercial and Self-pay** | | | |
| **Prescriber characteristics** | **Balanced group* n=35,545** | **Moderate^†^ n=72,614** | **High-risk prescribers^‡^ n=68** |
| **Specialty/Provider type** | **%** | **%** | **%** |
| Primary Care Physician |  |  |  |
| Emergency Physician | 14.5 | 31.8 | 14.7 |
| Other specialty | 39.7 | 12.1 | 0.0 |
| Physician Assistant/ Nurse Practitioner | 4.4 | 3.8 | 4.4 |
| Pain Physician | 30.1 | 19.2 | 10.3 |
| Surgeon | 0.2 | 4.2 | 61.8 |
| **Quartile Non-white or Hispanic** | 11.2 | 28.9 | 8.8 |
| First (most white) |  |  |  |
| Second | 4.3 | 4.3 | 2.9 |
| Third | 12.0 | 11.5 | 5.9 |
| Fourth (most minority) | 30.9 | 30.3 | 32.4 |
| **Overdose rate (quartile)** | 52.8 | 54.0 | 58.8 |
| First (lowest rate) |  |  |  |
| Second | 6.5 | 8.5 | 2.9 |
| Third | 29.3 | 34.1 | 36.8 |
| Fourth (highest rate) | 32.7 | 32.0 | 32.4 |
| **Urbanicity** | 31.5 | 25.3 | 27.9 |
| Urban |  |  |  |
| Rural | 87.1 | 83.1 | 92.6 |
| **Total Opioid per capita (tercile)** | 12.9 | 16.9 | 7.4 |
| First (lowest) |  |  |  |
| Second | 8.5 | 8.6 | 1.5 |
| Third | 40.7 | 37.7 | 30.9 |

^*^ Balanced prescribers: clinicians whose overall high-risk prescribing was less than the mean pooled rate among all in the dyad and for whom the probability that the rates of high-risk and difference between the rates of high-risk prescribing across payers are within a 20% confidence interval around zero

^†^ Moderate prescribers: clinicians for whom the rate difference in high risk prescribing between payers fell between 0 and the top 5^th^ or 0 and the bottom 5^th^ percentile of the distribution

^‡^ High-risk prescribers: clinicians whose overall high-risk prescribing was higher than mean pooled rate among all in the dyad

| **Appendix Table 2: Patient characteristics of episode overall and by prescriber group, by dyad** | | | | | | | | | |
| --- | --- | --- | --- | --- | --- | --- | --- | --- | --- |
| **Medicaid and Self-pay episodes treated by Medicaid and Self-pay providers (n=8,078,116)** | | | | | | | | | |
| Prescriber group | | | Balanced^*^ (n=3,214,328) | Moderate^†^ (n=4,406,515) | | Unbalanced^‡^ (n=450,514) | | High-risk^¶^ (n=6,759) | |
|  |  |  | % | % | | % | | % | |
| **Sex** | | |  |  | |  | |  | |
| Female | | | 61.2 | 62.3 | | 61.6 | | 53.1 | |
| Male | | | 38.8 | 37.7 | | 38.4 | | 46.9 | |
| **Age** | | |  |  | |  | |  | |
| 12-17 yo | | | 3.2 | 3.4 | | 3.6 | | 0.4 | |
| 18-25 | | | 17.9 | 15.3 | | 12.3 | | 3.0 | |
| 26-35 | | | 30.9 | 26.8 | | 22.9 | | 17.7 | |
| 36-45 | | | 21.3 | 20.3 | | 19.0 | | 25.2 | |
| 46-55 | | | 14.7 | 17.1 | | 18.6 | | 28.9 | |
| 56-65 | | | 9.0 | 13.0 | | 16.8 | | 21.8 | |
| 66+ | | | 3.0 | 4.2 | | 6.7 | | 3.0 | |
| **Medicaid and Commercial episodes treated by Medicaid and Commercial provider (n=20,148,616)** | | | | | | | | |  |
| Prescriber group | Balanced^*^ (n=6,822,656) | | | Moderate ^†^ (n=12,340,921^)^ | | Unbalanced^‡^ (n=963,362) | | High-risk^¶^ (n=21.677) |  |
|  | % | | | % | | % | | % |  |
| **Sex** |  | | |  | |  | |  |  |
| Female | 62.6 | | | 62.9 | | 61.0 | | 62.2 |  |
| Male | 37.4 | | | 37.1 | | 39.0 | | 37.8 |  |
| **Age** |  | | |  | |  | |  |  |
| 12-17 yo | 3.0 | | | 2.8 | | 2.3 | | 0.3 |  |
| 18-25 | 14.8 | | | 10.6 | | 7.6 | | 2.4 |  |
| 26-35 | 24.6 | | | 19.3 | | 16.0 | | 11.7 |  |
| 36-45 | 20.5 | | | 18.9 | | 18.2 | | 20.4 |  |
| 46-55 | 18.1 | | | 20.9 | | 22.6 | | 29.7 |  |
| 56-65 | 14.3 | | | 20.4 | | 24.3 | | 27.3 |  |
| 66+ | 4.7 | | | 7.0 | | 9.0 | | 8.4 |  |
| **Self-pay and commercial episodes treated by self-pay and commercial providers (n=16,676,807)** | | | | | | | | |  |
| Prescriber group | Balanced^*^ (n=5,036,772) | | | Moderate^†^ (n=10,705,578) | | Unbalanced^‡^ (n=918,764) | | High-risk^¶^ (n=15,693) |  |
|  | % | | | % | | % | | % |  |
| **Sex** |  | | |  | |  | |  |  |
| Female | 55.1 | | | 56.3 | | 55.1 | | 56.3 |  |
| Male | 44.9 | | | 43.7 | | 44.9 | | 43.7 |  |
| **Age** |  | | |  | |  | |  |  |
| 12-17 yo | 1.5 | | | 1.0 | | 1.5 | | 1.0 |  |
| 18-25 | 7.4 | | | 3.2 | | 7.4 | | 3.2 |  |
| 26-35 | 15.9 | | | 10.5 | | 15.9 | | 10.5 |  |
| 36-45 | 18.2 | | | 18.7 | | 18.2 | | 18.7 |  |
| 46-55 | 21.6 | | | 25.5 | | 21.6 | | 25.5 |  |
| 56-65 | 23.0 | | | 27.0 | | 23.0 | | 27.0 |  |
| 66+ | 12.5 | | | 14.0 | | 12.5 | | 14.0 |  |

^*^ Balanced prescribers: clinicians whose overall high-risk prescribing was less than the mean pooled rate among all in the dyad and for whom the probability that the rates of high-risk and difference between the rates of high-risk prescribing across payers are within a 20% confidence interval around zero

^†^ Moderate prescribers: clinicians for whom the rate difference in high risk prescribing between payers fell between 0 and the top 5^th^ or 0 and the bottom 5^th^ percentile of the distribution

^‡^ Unbalanced: Clinicians whose prescribing rate difference in prescribing between insurances was either in the top or bottom 5^th^ percentile

^¶^High-risk prescribers: clinicians whose overall high-risk prescribing was higher than mean pooled rate among all in the dyad

| **Appendix Table 3: Sensitivity analysis using only 90 MME as High-risk prescribing: Adjusted Odds Ratio^*^ of Characteristics of Unbalanced Prescribers vs. Other Prescribers^†^** | | | |
| --- | --- | --- | --- |
|  | **Medicaid-Self-Pay Dyad** | **Medicaid-Commercial Dyad** | **Commercial-Self-pay Dyad** |
|  | **aOR**^‡^ **(95% CI)** | **aOR**^‡^ **(95% CI)** | **aOR**^‡^ **(95% CI)** |
| **Specialty/Provider type** |  |  |  |
| Primary Care Physician | Ref | Ref | Ref |
| Emergency Physician | **0.3 (0.26-0.36)** | **0.4 (0.31-0.41)** | **0.5 (0.43-0.58)** |
| Other specialty | 1 (0.87-1.24) | **1.8 (1.61-1.94)** | **1.4 (1.24-1.54)** |
| Physician Assistant/ Nurse Practitioner | **0.7 (0.6-0.75)** | **0.8 (0.74-0.86)** | **0.6 (0.54-0.66)** |
| Pain Physician | **1.4 (1.14-1.61)** | **1.7 (1.52-2.01)** | **1.6 (1.44-1.83)** |
| Surgeon | **1.7 (1.54-1.89)** | **0.8 (0.78-0.91)** | **0.8 (0.79-0.91)** |
| **Quartile Non-white or Hispanic** |  |  |  |
| First | Ref | Ref | Ref |
| Second | 1 (0.77-1.25) | 0.9 (0.76-1.06) | 0.9 (0.78-1.11) |
| Third | 1.2 (0.99-1.56) | 1.1 (0.95-1.34) | 1 (0.89-1.25) |
| Fourth | 1.3 (1.04-1.71) | **1.5 (1.27-1.83)** | **1.2 (1.01-1.48)** |
| **Overdose rate (quartile)** |  |  |  |
| First | Ref | Ref | Ref |
| Second | 0.9 (0.74-1.06) | 1.1 (0.89-1.23) | 1 (0.84-1.13) |
| Third | 1 (0.84-1.25) | 1.1 (0.95-1.37) | 1.1 (0.89-1.27) |
| Fourth | 1 (0.77-1.19) | **1.3 (1.08-1.61)** | 1.1 (0.86-1.3) |
| **Urbanicity** |  |  |  |
| Urban | Ref | Ref | Ref |
| Rural | **0.7 (0.65-0.84)** | 1 (0.95-1.16) | 1 (0.91-1.12) |
| **Total Opioid per capita (tercile)** |  |  |  |
| First | Ref | Ref | Ref |
| Second | 1.1 (0.94-1.3) | 1.2 (0.97-1.37) | 1 (0.86-1.11) |
| Third | **1.3 (1.1-1.56)** | **1.4 (1.15-1.64)** | 1.1 (0.96-1.27) |
| **Minimum patient volume per prescriber^‡^** | 0.1 (0.08-0.1) | **0.2 (0.16-0.19)** | **0.1 (0.11-0.14)** |

* Logistic regression models also included state fixed-effects. Standard errors were clustered at the county level.

† Comparison group includes balanced, moderate, and high-risk prescribers

‡ Log-transformed minimum number of patients across 2 payers of each provider

Odds ratios that are statistically signification at p <.05 are bolded
